# Communicating with people with hearing loss: COVID-19 and beyond

**DOI:** 10.3399/BJGPO.2020.0174

**Published:** 2021-01-13

**Authors:** Devina Maru, Jack Stancel-Lewis, Graham Easton, William EJ Leverton

**Affiliations:** 1 National Clinical Champion for Deafness and Hearing Loss, Royal College of General Practitioners, London, UK; 2 GP Speciality Registrar, Greenwich, London, UK; 3 Healthcare Science Fellow (Audiology), Medical Directorate, NHS England and NHS Improvement, London, UK; 4 Professor of Clinical Communication Skills, Head of Clinical and Communication Skills Unit, Barts and the London School of Medicine and Dentistry, London, UK; 5 Royal College of General Practitioners Clinical Advisor, Devon, UK

**Keywords:** Hearing Loss, ENT, Consultation skills, COVID-19, Family Medicine, Primary Health Care, General Practice, Deafness

Hearing health and effective communication is more important than ever. Since the COVID-19 pandemic resulted in wide scale social distancing in the UK, advice from the government has been to limit contact with others, and for older people and those with underlying health conditions to shield.^1^ This has dramatically exacerbated social isolation within this population. The widespread use of face coverings has impeded communication by blocking the listener’s view of the lips.^2^ By forcing the wearing of masks and increasing the use of telemedicine, the COVID-19 pandemic has focused the minds of healthcare professionals on communicating with people with hearing loss.^3,4^ In this article we offer practical tips and resources for busy GPs, including the new Royal College of General Practitioners (RCGP) Toolkit for Deafness and Hearing Loss.

Hearing loss is one of the commonest disabilities seen in primary care. Between 20% and 50% of primary care appointments are related to ear, nose, and throat (ENT) conditions.^5^ It affects one in six of the UK population; one in two over the age of 70. Hearing loss is associated with poor social interaction, isolation, depression and anxiety, reduced quality of life, and increased risk of dementia. Treating hearing loss may be one of the most cost-effective ways of reducing someone’s risk of dementia.^6^ The wearing of a face mask may result in unintended consequences such as social isolation and poor mental wellbeing. This may predispose a large section of society to mask misery.^7^

Acquired, age-related hearing loss occurs gradually. Your patients with deteriorating hearing may have noticed subtle clues such as the gradual fading of birdsong, increasing the volume on the television, muffled speech, or difficulty hearing in background noise. Often, it is the person’s relatives or friends that notice changes in their loved one’s hearing. The author Helen Keller pointed out that hearing loss cuts you off from people, and those with the condition will confirm this hasn’t changed despite the use of increasingly sophisticated hearing aids. The hearing mechanisms themselves are damaged, reducing the value of amplification, so patients must increase their reliance on other communication cues such as lip movements, body language, and understanding of context. The extra processing required results in both slower communication and fatigue.

Communication is more than just spoken language. Communication between humans occurs most successfully when all the elements of body language, speech, and hearing are deployed. Consider wearing earplugs and placing yourselves in the shoes of this large group of your patients. Good communication techniques and awareness encourages inclusivity which can address the isolation and marginalisation of people with hearing loss. Knowing how to communicate well will increase patient satisfaction and, importantly, lead to vastly improved communication (and trust) between healthcare professionals and patients.

## Improving access

For some time, patients (and more recently GPs) have identified a lack of GP training in the necessary skills to communicate effectively with people who have hearing loss.^8^ The *Access All Areas* report collected the experiences of people with hearing loss accessing health care, including contacting and visiting their GP surgery. Responders with varying levels of hearing loss completed the survey and the findings were eye-opening.^9^ GP appointments were made in person by 47% of responders, yet only 14% identified this as their preferred method of contact. During GP consultations, 49% of responders said that their GP surgery had a visual display screen, but one in seven responders had missed an appointment because they had missed being called from the waiting room. NHS England estimated that the cost of people who are Deaf or have hearing loss missing NHS appointments could be as high as £15 million every year. After attending their appointment, more than a quarter (28%) of patients were unclear about their diagnosis and the health advice they had been given.

Access to primary care for the Deaf or hearing impaired can be stressful, from arranging an appointment by telephone, to the fear of not hearing what is said in the consultation. In a recent survey by Royal National Institute for the Deaf (RNID), more than 70% of responders said they *’did not feel confident that their communication needs would be met during a remote appointment’* and over half admitted they had *’put off’* seeking advice from their GP because of remote care, meaning they were at risk of missing out on vital medical care. An awareness of the potential barriers to effective communication and the implementation of some simple strategies will dramatically improve communication with your Deaf and hard of hearing patients. The RCGP, in conjunction with the charity RNID and NHS England and NHS Improvement, have developed a toolkit of learning materials to fill this lacuna (see Box 1).

Box 1 **Education Resources for Primary Care**

- *RCGP Deafness and Hearing Loss Toolkit https://www.rcgp.org.uk/clinical-and-research/resources/toolkits/deafness-and-hearing-loss-toolkit.aspxhttps://www.rcgp.org.uk/hearingloss*
- *RCGP Accredited Deaf Awareness Online Training Course* *https://www.rcgp.org.uk/learning/rcgp-educational-accreditation-for-education-providers/accredited-activities.aspx*
- *The RCGP Curriculum Topic Guides: Ear, Nose and Throat, Speech and Hearing. London, Royal College of General Practitioners: 2019. pp 133–141. https://www.rcgp.org.uk/-/media/Files/GP-training-and-exams/Curriculum-2019/Curriculum-Topic-Guides-300819.ashx?la=en*

Designed to bring together resources for healthcare professionals, it includes case studies and teaching material suitable for use in training schemes as well as in the GP surgery. In addition, the RCGP Core Curriculum on hearing loss has been expanded and brought up to date. The GP curriculum now stresses the importance of early intervention, effective communication, and improved access for people who are Deaf or have hearing loss. The RCGP has also accredited a Deaf awareness online course for doctors, developed by UCL Deafness Cognition and Language Research Centre. This 2-hour self-directed course will help you understand the communication needs of Deaf and hearing-impaired patients. It provides practical workplace strategies to help meet the Accessible Information Standard. There are videos of patients' and doctors’ real-life experiences to make the learning relevant and engaging.

## Remote consulting

Communication is a key skill for our specialty (see Box 2). Remember that people who are Deaf or have hearing loss have individual communication needs, and you should ask the person concerned how best to communicate with them. Not every tip below will be appropriate for every person. Visual tips to be aware of: people might choose to wear the Sunflower or Hidden Disability Lanyard, or homemade badges to highlight their needs.^10^ They may also carry exemption cards to show that they do not need to wear a face covering, or to highlight where they might need others to lower theirs.

Box 2 **Key tips for communicating with people who have hearing loss during video consultation appointments include:**

**Before you start**

- Exclude background noise
- Face the camera
- Ensure your face is well lit and they can see your lips
- Check they are wearing their hearing aids

**During the consultation**

- Speak clearly, use plain language, do not shout
- Speak at a normal pace, not too slow or too fast
- Use normal lip movements, facial expressions, gestures
- Tell he patient if live speech to text is available

**Confirmation**

- If asked to repeat something more than once, use different words
- OR write things down: pen on paper, text on screen, or whiteboard
- Ask if the patient understood what you said; offer to repeat if they did not

With the introduction of remote consulting, it is essential to equip GPs with the tools and knowledge to assist in communicating with patients who have a hearing impairment. Instead of using the telephone, which relies on sound alone, where possible use video conferencing tools and add live captioning through video conferencing software. Utilise *Relay UK* ^11^ for people with hearing loss; use video relay services (VRS), such as *Interpreter Now*, for British Sign Language (BSL) users. *BSL Health Access* ^12^ provides immediate, on-demand access to BSL interpreters for communication with Deaf people free of charge during this pandemic. Access to BSL interpreters takes place through two methods: VRS, where a BSL interpreter relays information over a telephone call between a BSL user and the hearing person receiving or making the call; and video remote interpreting (VRI), where a remote interpreter is used to facilitate communication with a Deaf and hearing person in the same location. Additionally, speech-to-text apps are very useful during consultations; these are widely available and many are free to download. It converts the clinician’s voice into written text which can be read by the patient during the consultation.

The RCGP Hearing Loss Toolkit will improve GPs ability to communicate with this large group of patients. We will all benefit; hearing loss is one condition from which almost every one of us will eventually suffer.
